# Maternal Weight Gain in Early Pregnancy with Healthy Live Offspring: Based on the China Birth Cohort Study

**DOI:** 10.3390/nu16132154

**Published:** 2024-07-06

**Authors:** Jingjing Wang, Simin Zhang, Qiao Li, Xiaowei Xiong, Qingqing Wu

**Affiliations:** 1 Clinical Trial Institution Office, Beijing Obstetrics and Gynecology Hospital, Capital Medical University, Beijing Maternal and Child Health Care Hospital, Beijing 100026, China; 2 Department of Ultrasound, Beijing Obstetrics and Gynecology Hospital, Capital Medical University, Beijing Maternal and Child Health Care Hospital, Beijing 100026, China

**Keywords:** Chinese pregnant women, early pregnancy, maternal weight gain, BMI, birth cohort

## Abstract

Background: Research on maternal weight gain in early pregnancy with healthy live offspring is lacking for Chinese women. Based on the China birth cohort study (CBCS), we aimed to explore maternal weight gain in different groups. Methods: Singleton pregnancies of 6 ^+ 0^~13 ^+ 6^ weeks of gestation from the CBCS were considered, not including missing data or outliers, those lost at follow-up, or those with non-typical conditions of the offspring. Maternal first-trimester weight and body mass index (BMI) gain was considered as the early pregnancy weight minus the pre-pregnancy weight. Using Pearson’s or Spearman’s correlation and linear regression models to explore the relationship between maternal weight and BMI gain and gestational age (GA), stratified and sensitivity analyses were carried out to identify the study’s robustness. Results: There were 25,292 singleton pregnancies with healthy live offspring who were ultimately enrolled, and there was a linear correlation between GA and maternal weight gain (=0.55 + 0.05 × GA (weeks), *p* < 0.001, r^2^ = 0.002) and BMI change (=0.21 + 0.02 × GA (weeks), *p* < 0.001, r^2^ = 0.002). The association remained robust in the stratified and sensitivity analyses of the subgroups. Conclusions: Although the association between GA and maternal pre-pregnancy weight and BMI gain is weak, a slight correlation was shown, especially in pregnant women with a typical or low pre-pregnancy BMI, Han ethnicity, moderate levels of physical activity, natural conception, and folic acid (FA) and/or multivitamin supplementation.

## 1. Background

Gestational weight gain (GWG) has become one of the hottest issues in modern obstetrics [1,2]. It has been identified as a strong and potentially controllable predictor of pregnancy and long-term health outcomes in women and infants, and is associated with various adverse outcomes [2,3,4,5,6]. An expert review [3] showed excessive GWG existed in about 48% of pregnant individuals in the United States and is associated with an increased risk of maternal and fetal complications. Analyzing the metabolomic profiles of children at birth, 6 months, and 12 months of age with maternal GWG, Guixeres-Esteve et al. [4] suggested that some metabolic shifts occurred during the first 12 months of life, which GWG may have modulated. Fritsche et al. [5] analyzed the relationship between maternal GWG and offspring autonomic nervous system (ANS) function in 2-year-old children and suggested that GWG has a critical impact on the development of the ANS. Excessive GWG may also increase the risk of neurodevelopmental disorders in offspring [6]. In addition, many factors affect GWG during pregnancy, such as behavioral, obstetric, and anthropometry characteristics [7]. The role of GWG should not be underrated [8].

The U.S. Institute of Medicine (IOM) provided recommendations on the total GWG (a gain of 12.5–18 kg for women with a pre-pregnancy body mass index (BMI) < 18.5, 11.5–16 kg for those with a BMI of 18.5–24.9, 7–11 kg for a BMI of 25–29.9, and 5–9 kg for a BMI ≥ 30) as well as for rates of GWG (0.44–0.58, 0.35–0.50, 0.23–0.33, and 0.17–0.27 kg/week, respectively) during the second and third trimesters based on BMI [9]. A systematic review and meta-analysis included 23 studies and found that, compared to GWG within the IOM-recommended levels, those with greater than or less than the guideline recommendations were associated with a higher risk of adverse maternal and infant outcomes [10]. Although the IOM guidelines are not universally implemented, they provide clinicians with a basis for practice [11,12]. However, some studies have reported inconsistent results on the link between GWG in particular BMI categories and adverse pregnancy outcomes, and what constitutes adequate GWG is relatively confusing [1,13,14,15].

Most studies have focused on the total GWG and rates of GWG during the second and third trimesters. Maternal weight gain in early pregnancy is also essential to women and infants. Calculating maternal weight gain for periods of <20 weeks, 21–29 weeks, and  ≥30 weeks, Young et al. [16] analyzed 864 mother–child pairs in Vietnam and found that weight gain in the less-than-20-weeks group had the most remarkable association with postpartum weight retention and maternal (β 0.67, 95% CI 0.07 to 0.87) or child (β 0.42, 95% CI 0.15 to 0.69) percentage of body fat at 6 to 7 years. Additionally, our previous study, based on the China birth cohort study (CBCS), found that excessive weight gain in early pregnancy may be a potential predictor of fetal congenital heart disease (CHD) [15]. We hypothesized that maternal weight and BMI gain during early pregnancy were associated with gestational age (GA). The potential association might differ by maternal and fetal status, and little research has explored this association in cases with healthy live offspring based on a multicenter cohort study of Chinese women. Therefore, our study aimed to describe maternal weight and BMI gain with healthy live offspring in early pregnancy and analyze its relationship with GA, adjusted for maternal age, maternal pre-pregnancy BMI, morning sickness, and other confounding factors.

## 2. Methods

### 2.1. Study Design and Data Sources

**Figure 1:**
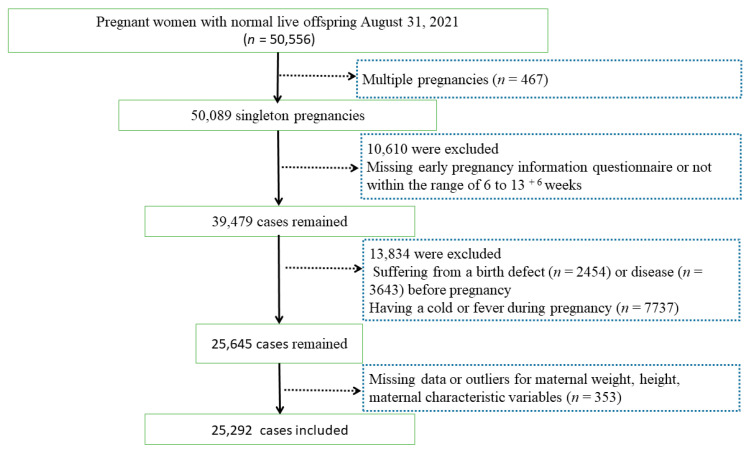
Flowchart of the study’s participant selection. This study ultimately included 25,292 singleton pregnancies.

The CBCS [17], a prospective, longitudinal, multi-center birth cohort study in China, started in November 2017, and data on pregnant women with healthy live offspring (*n* = 50,556) were extracted from the electronic system of the CBCS on 31 August 2021. The exclusion criteria included multiple pregnancies (*n* = 467); a missing early pregnancy information questionnaire or not being within the range of 6 to 13 ^+ 6^ weeks (*n* = 10,610); suffering from a birth defect (*n* = 2454) or disease (3643) before pregnancy, such as congenital heart defect, cleft lip and palate, polydactyly, heart disease, hypertension, diabetes, hepatitis B, thyroid disease, genital inflammation, periodontal disease; having a cold or fever during pregnancy (*n* = 7737); missing data or outliers for maternal weight or height (*n* = 305); missing maternal characteristic variables (*n* = 48), such as age, ethnicity, smoking or drinking status. Thus, 25,292 singleton pregnancies were ultimately included (Figure 1). The research was approved by the Ethics Committee of Beijing Obstetrics and Gynecology Hospital, Capital Medical University (Reference No. 2018-KY-003-02).

### 2.2. Data Collection and Measurements

In the CBCS, women were enrolled in early pregnancy and completed the baseline questionnaire by themselves during early pregnancy. Participation in the study was voluntary, and informed consent was obtained in writing. Their weights were accurately measured using an electronic scale (BW-150; UWE, Beijing, China), with the participants wearing light clothes, no shoes, and empty pockets [18]. This study collected the following information: (a) Demographic characteristics, including investigation date, maternal birth date, weight, height, ethnicity, education, family income, and occupation. (b) Current pregnancy information, including folic acid (FA) and multivitamin use in early/pre-pregnancy, last menstrual period, conception mode, and parity. (c) Lifestyle behaviors, including maternal alcohol consumption, smoking, and secondhand smoke exposure. Table 1 shows the calculation and classification of variables.

### 2.3. Statistical Analysis

Data calculations and statistical analyses were performed using IBM SPSS Statistics V.25 and SAS V.9.4 software. A *p* value of less than 0.05 was considered statistically significant. Quantitative data were expressed by the mean and standard deviation or the median and quartile range. As appropriate, the *t*-test, ANOVA test, Wilcoxon test, or Kruskal–Wallis H test was performed to calculate differences in the maternal weight and BMI gain among the maternal characteristics. The reference values for the 5th, 10th, 25th, 50th, 75th, 90th, and 95th percentiles for seven gestational periods (from 7 to 13 weeks) were calculated. Pearson’s correlation, Spearman’s correlation, and linear regression models were used to explore the relationship between maternal weight and BMI change and GA. Stratification was carried out to identify the robustness of the association of the maternal weight and BMI change in early pregnancy with GA in subgroups, including age (<35 years or ≥35 years), BMI (normal, low, overweight, or obesity), ethnicity (Han or Minority), family income (<200,000 or ≥200,000 CNY/year), physical activity (moderate, light, or active), educational level (college or university, high school or below, or postgraduate), parity (multipara or nullipara), smoking or drinking status (yes or no), morning sickness (yes or no), and FA and/or multivitamin supplementation (yes or no). A sensitivity analysis was performed in cases with normal or majority maternal status.

## 3. Results

### 3.1. Characteristics

Among all participants, 25,292 singleton pregnancies with healthy live offspring were ultimately enrolled, of which 1797 (7.1%), 3226 (12.8%), 2992 (11.8%), 2183 (8.6%), 1705 (6.7%), 2716 (10.7%), 7082 (28.0%), and 3591 (14.2%) cases answered the questionnaire in the periods of 6, 7, 8, 9, 10, 11, 12, and 13 gestational weeks, respectively. The mean and median ages were 29.6 ± 4.3 years and 29 (27, 32) years, respectively. The means of the maternal weight and BMI gain in early pregnancy were 1.0 ± 2.4 kg and 0.39 ± 0.92 kg/m^2^, while the medians were 1.0 (0.0, 2.0) kg and 0.37 (0.00, 0.78) kg/m^2^, respectively. Table A1 and Table A2 in the Appendix A show the percentiles of weight gain and BMI change in different gestational weeks. Table 2 presents the significant differences in maternal weight and BMI change among age, BMI, ethnicity, income, educational level, parity, smoking or drinking status, morning sickness, FA and/or multivitamin supplementation, and GA (all with *p*-values < 0.05). 

### 3.2. Relationship between Maternal Weight and BMI Gain and GA

**Figure 2:**
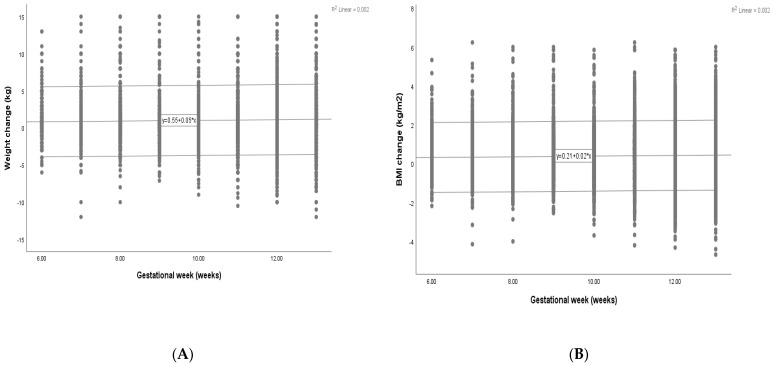
Plots of maternal weight and BMI gain with advancing GA. (**A**) The plot of maternal weight gain with GA. (**B**) The plot of maternal BMI gain with GA. The middle line is the linear regression, and the lines on either side represent the 95% confidence intervals of the individual. The * in the formula represents the multiplication sign. GA, gestational age; BMI, body mass index.

Maternal weight and BMI gain demonstrated linear correlations with GA (*p* = 0.001, <0.001). The scatter plot of the maternal weight and BMI gain shows progressive changes with advancing gestation (Figure 2). The mean maternal weight change increased slightly from 0.85 kg in 6 weeks to 1.20 kg in 13 weeks (weight gain = 0.55 + 0.05 × GA (weeks), *p* < 0.001, r^2^ = 0.002). Similarly, the maternal BMI change shows a linear increase (BMI change = 0.21 + 0.02 × GA (weeks), *p* < 0.001, r^2^ = 0.002). 

### 3.3. Stratified Analyses

The association of the maternal weight and BMI change in early pregnancy with GA was also found in subgroups, including age (<35 years or ≥35 years), BMI (normal or low), Han ethnicity, family income (<200,000 or ≥200,000 CNY/year), educational level (college or university, high school or below, and postgraduate), moderate levels of physical activity, parity (multipara or nullipara), smoking or drinking status (yes or no), natural conception, morning sickness (yes or no), and with FA and/or multivitamin supplementation (Table 3).

### 3.4. Sensitivity Analysis

Cases with normal or majority maternal status, including maternal normal BMI, Han ethnicity, natural conception, with FA and/or multivitamin supplementation, and without smoking or drinking status or morning sickness were selected, and 7431 cases were included to perform sensitivity analysis. The association of maternal weight and BMI gain in early pregnancy with GA was still found (Table 4). The mean maternal weight gain increased slightly from 0.76 kg at 6 weeks to 1.32 kg at 13 weeks, while the maternal BMI increased from 0.28 kg/m^2^ to 0.49 kg/m^2^.

## 4. Discussion

### 4.1. Main Findings of This Study

This study included a relatively large sample size of 25,292 singleton pregnancies with healthy live offspring based on the China birth cohort study, allowing us to explore the potential association of maternal weight and BMI change in early pregnancy with GA in subgroups, including maternal demographic characteristics, current pregnancy information, and lifestyle behaviors. There was a slight correlation between GA and maternal weight and BMI gain. The association remained robust especially in pregnant women with typical or low pre-pregnancy BMI, Han ethnicity, moderate levels of physical activity, natural conception, and with FA and/or multivitamin supplementation, regardless of maternal age, family income, educational level, parity, morning sickness, smoking or drinking status. Furthermore, the associations of maternal weight and BMI gain in early pregnancy with GA were also shown in the normal or majority maternal status.

### 4.2. Possible Reasons and Comparison with Previous Studies

A retrospective study [19] with 9075 singleton pregnant women in China found that the 2009 IOM guideline was more suitable for pregnant women with inadequate GWG. Meanwhile, another retrospective cohort study [20] comprising 20,593 singleton pregnant women in Beijing, China, found that the IOM guidelines were suitable for Chinese women with underweight pre-pregnancy BMI. Maternal weight gain in early pregnancy is also associated with the outcomes of pregnant women and fetuses. Gaillard et al. [21] analyzed 5,908 mothers and their children from a population-based prospective cohort study and found that higher weight gain in early but not in mid or late pregnancy was associated with an adverse cardio-metabolic profile in offspring. Additionally, Young et al. [22] used secondary data with 1436 cases from a randomized controlled trial in Vietnam and found that compared to ≥ 30 weeks’ gestation, 1 SD increase in maternal conditional weight gain in the first 20 weeks had three times the influence on birth weight and was associated with a 43% reduction in small for gestational age (SGA) risk (OR 0.57, 95% CI 0.46 to 0.70). As we all know, maternal weight gain is associated with GA and could be influenced by multiverse factors, such as demographic characteristics, intervention, breastfeeding practices, diet, and physical activity.

Nausea and vomiting during pregnancy (NVP), also called morning sickness, usually begins at 4–6 weeks of gestation, peaks at 8–12 weeks, and often disappears naturally at mid-pregnancy, affecting 70% of pregnant women [23,24,25]. The risk of severe NVP may be influenced by a protein released by fetal cells in the placenta [26], which could affect maternal weight gain. The association of maternal weight and BMI change in early pregnancy with GA was found in both those with morning sickness and those without. Unfortunately, the lack of information on cases suffering from hyperemesis gravidarum and on the second and third trimesters limits the study.

Previous guidelines and recommendations have suggested that appropriate maternal weight gain differs by the pre-pregnancy BMI range [9,11]. Ukah et al. explored the association between GWG and severe adverse birth outcomes in the US by calculating the optimal, low, and excess weight gain in each BMI category [27]. This study also found an association of maternal weight and BMI change in early pregnancy with GA in typical or low BMI subgroups but not in the overweight or obesity group. One possible reason may be that women who are overweight or obese before pregnancy are more focused on their weight management during pregnancy. The other reason may be that 73.5% of participants had typical BMI, and those with overweight (10.6%) or obesity (2.1%) were lower. Additionally, pregnant women with underweight, overweight, or obese status may be more coached by their doctors to gain or lose weight.

This study also found the association of maternal weight and BMI change in early pregnancy with GA in the multipara and nullipara subgroups. Cohen et al. conducted a population-based observational study in Canada and found that women whose firstborn experienced certain adverse perinatal events gained more weight and were more likely to transition to a higher BMI category in their subsequent pregnancy [28]. Unfortunately, the details of the firstborn in the multipara group were lacking in this study, which may lead to a bias. 

Because the cases with abnormal BMI, minority ethnicity, assisted reproduction, without FA and/or multivitamin supplementation, and smoking or drinking status were relatively small in this study, and because morning sickness could affect maternal weight gain in early pregnancy, a sensitivity analysis was carried out to identify the study’s robustness by selecting cases with normal or majority maternal status. An association of maternal weight and BMI change in early pregnancy with GA was still found.

### 4.3. Limitations of the Study

Several limitations must be acknowledged. First, although this study included a relatively large sample size of singleton pregnancies with healthy live offspring based on the CBCS, not all confounders affecting maternal weight were included, and some related influence on these unknown factors cannot be ruled out. Second, the maternal weight in the periods of 6-14 gestational weeks was not measured continuously, and there were no data on the weight gain during the second and third trimesters. Third, since the last follow-up was performed after delivery, the healthy live-born offspring defined in this study may also have conditions such as disease or defects in later infancy. Fourth, GA was calculated as the first date of the last menstrual cycle in this study and not determined by ultrasound, which may lead to bias. Furthermore, many essential data (lifestyle behaviors, including maternal alcohol consumption, smoking, and exposure to passive smoking) were collected through questionnaires, and there may be information bias. All of these factors may lead to biased estimates and indicate that the validation of our results is necessary, especially in longitudinal data with continuous maternal weight measurement and prolonged follow-up time of offspring.

## 5. Conclusions

Although the association of GA and maternal pre-pregnancy weight and BMI gain is weak, a slight correlation was shown, especially in pregnant women with a typical or low pre-pregnancy BMI, Han ethnicity, moderate levels of physical activity, natural conception, and with FA and/or multivitamin supplementation. However, this study does not suggest that weight gain during early pregnancy is better than weight loss. This finding may be helpful for determining the appropriate range of maternal weight gain in early pregnancy with healthy live offspring in China and other countries.

## Figures and Tables

**Table 1:** The calculation and classification of variables.

Variables	Calculation and Classification
Maternal weight gain (kg)	Weight in early pregnancy (kg) minus that in pre-pregnancy (kg)
BMI	Weight (kg) divided by height (m) squared #
Gestational age (weeks)	Survey date minus date of menstruation
BMI gain (kg/m^2^)	BMI (kg/m^2^) in early pregnancy minus that in pre-pregnancy (kg/m^2^)
Maternal age (years)	Date of survey minus date of birth
Maternal physical activity	Active (e.g., farmer or manual laborer); moderate (e.g., teacher, salesperson, or clerk); light (e.g., unemployed).

Note: BMI, body mass index. # Based on the World Health Organization (WHO) criteria, BMI was categorized as obesity (BMI ≥ 30.0 kg/m^2^), overweight (25.0–29.9 kg/m^2^), normal weight (18.5–24.9 kg/m^2^), and low (<18.5 kg/m^2^).

**Table 2:** Maternal pre-pregnancy weight change in different groups.

Groups	*n*	Weight Change (kg)		*p*	BMI Change (kg/m^2^)		*p*
		Mean ± SD	Median (Q1, Q3)		Mean ± SD	Median (Q1, Q3)	
Total	25,292	1.0 ± 2.4	1.0 (0.0, 2.0)	-	0.39 ± 0.92	0.37 (0.00, 0.78)	-
Maternal age (years)				<0.001			<0.001
<35	22,045	1.0 ± 2.4	1.0 (0.0, 2.0)		0.38 ± 0.93	0.37 (0.00, 0.78)	
≥35	3247	1.2 ± 2.3	1.0 (0.0, 2.0)		0.47 ± 0.86	0.38 (0.00, 0.80)	
Maternal BMI (kg/m^2^)				<0.001			<0.001
Normal	18,599	1.0 ± 2.4	1.0 (0.0, 2.0)		0.38 ± 0.92	0.37 (0.00, 0.78)	
Low	3473	1.3 ± 2.2	1.0 (0.0, 2.0)		0.48 ± 0.84	0.38 (0.00, 0.78)	
Overweight	2688	0.9 ± 2.6	1.0 (0.0, 2.0)		0.34 ± 0.97	0.37 (0.00, 0.78)	
Obesity	532	0.9 ± 3.4	0.0 (0.0, 2.0)		0.34 ± 1.31	0.00 (0.00, 0.83)	
Maternal ethnicity				0.027			0.041
Han	23,630	1.0 ± 2.4	1.0 (0.0, 2.0)		0.39 ± 0.93	0.37 (0.00, 0.78)	
Minority	1662	1.0 ± 2.2	1.0 (0.0, 2.0)		0.35 ± 0.84	0.36 (0.00, 0.76)	
Family income, CNY/year				<0.001			<0.001
<200,000	18,004	1.1 ± 2.6	1.0 (0.0, 2.0)		0.41 ± 0.98	0.37 (0.00, 0.80)	
≥200,000	7288	0.9 ± 2.0	1.0 (0.0, 2.0)		0.34 ± 0.74	0.35 (0.00, 0.73)	
Maternal educational level				<0.001			<0.001
College/university	17,502	1.0 ± 2.4	1.0 (0.0, 2.0)		0.39 ± 0.92	0.37 (0.00, 0.78)	
High school or below	5148	1.1 ± 2.6	1.0 (0.0, 2.1)		0.43 ± 1.02	0.38 (0.00, 0.87)	
Postgraduate	2642	0.8 ± 1.8	1.0 (0.0, 2.0)		0.32 ± 0.70	0.34 (0.00, 0.70)	
Maternal physical activity				0.429			0.318
Moderate	18,775	1.0 ± 2.2	1.0 (0.0, 2.0)		0.37 ± 0.85	0.37 (0.00, 0.78)	
Light	6190	1.2 ± 2.9	1.0 (0.0, 2.0)		0.43 ± 1.11	0.36 (0.00, 0.82)	
Active	327	1.2 ± 2.5	1.0 (0.0, 2.0)		0.47 ± 0.97	0.38 (0.00, 0.83)	
Parity				0.002			0.021
Multipara	12,576	1.0 ± 2.3	1.0 (0.0, 2.0)		0.37 ± 0.90	0.37 (0.00, 0.78)	
Nullipara	12,716	1.1 ± 2.5	1.0 (0.0, 2.0)		0.41 ± 0.94	0.37 (0.00, 0.78)	
Maternal smoking				<0.001			<0.001
No	24,799	1.0 ± 2.4	1.0 (0.0, 2.0)		0.38 ± 0.92	0.37 (0.00, 0.78)	
Yes	493	1.7 ± 2.6	1.4 (0.0, 3.0)		0.64 ± 0.96	0.52 (0.00, 1.29)	
Maternal secondhand smoke exposure				<0.001			<0.001
No	22,251	1.0 ± 2.4	1.0 (0.0, 2.0)		0.38 ± 0.92	0.37 (0.00, 0.78)	
Yes	3041	1.1 ± 2.4	1.0 (0.0, 2.0)		0.43 ± 0.92	0.39 (0.00, 0.81)	
Maternal drinking				0.001			<0.001
No	24,590	1.0 ± 2.4	1.0 (0.0, 2.0)		0.39 ± 0.92	0.37 (0.00, 0.78)	
Yes	702	0.9 ± 2.5	1.0 (0.0, 2.0)		0.36 ± 0.95	0.36 (0.00, 0.78)	
Mode of conception				0.425			0.435
Natural conception	24,412	1.0 ± 2.4	1.0 (0.0, 2.0)		0.38 ± 0.93	0.37 (0.00, 0.78)	
Assisted reproduction	880	1.2 ± 2.5	1.0 (0.0, 2.4)		0.38 ± 0.93	0.37 (0.00, 0.78)	
Morning sickness				<0.001			<0.001
No	13,994	1.1 ± 2.2	1.0 (0.0, 2.0)		0.42 ± 0.84	0.38 (0.00, 0.78)	
Yes	11,298	0.9 ± 2.6	1.0 (0.0, 2.0)		0.34 ± 1.01	0.35 (0.00, 0.78)	
FA and/or MV supplementation				0.001			0.003
No	673	0.7 ± 2.6	0.5(0.0, 2.0)		0.28 ± 0.99	0.20 (0.00, 0.78)	
Yes	24,619	1.0 ± 2.4	1.0 (0.0, 2.0)		0.39 ± 0.92	0.37 (0.00, 0.78)	
Gestational age (weeks)				<0.001			<0.001
6~	1797	0.8 ± 1.6	0.5 (0.0, 1.5)		0.32 ± 0.65	0.21 (0.00, 0.53)	
7~	3226	0.9 ± 1.7	0.9 (0.0, 2.0)		0.34 ± 0.65	0.33 (0.00, 0.68)	
8~	2992	1.0 ± 2.1	1.0 (0.0, 2.0)		0.37 ± 0.78	0.35 (0.00, 0.74)	
9~	2183	1.0 ± 2.2	1.0 (0.0, 2.0)		0.37 ± 0.84	0.36 (0.00, 0.75)	
10~	1705	0.9 ± 2.5	1.0 (0.0, 2.0)		0.34 ± 0.96	0.35 (0.00, 0.76)	
11~	2716	1.0 ± 2.6	1.0 (0.0, 2.0)		0.38 ± 1.00	0.38 (0.00, 0.80)	
12~	7082	1.1 ± 2.7	1.0 (0.0, 2.0)		0.40 ± 1.03	0.38 (0.00, 0.84)	
13~	3591	1.3 ± 2.9	1.0 (0.0, 3.0)		0.50 ± 1.00	0.40 (0.00, 1.06)	

Abbreviations: BMI, body mass index; FA, folic acid; MV, multivitamins.

**Table 3:** The association of maternal weight and BMI gain in early pregnancy with GA in various subgroups.

Groups	*n*	Weight Change (kg)	*p*	BMI Change (kg/m^2^)	*p*
Maternal age (years)					
<35	22,045	=0.57 + 0.04 × GA	<0.001	=0.21 + 0.02 × GA	<0.001
≥35	3247	=0.28 + 0.10 × GA	<0.001	=0.10 + 0.04 × GA	<0.001
BMI (kg/m^2^)					
Normal	18,599	=0.51 + 0.05 × GA	<0.001	=0.19 + 0.02 × GA	<0.001
Low	3473	=0.46 + 0.08 × GA	<0.001	=0.16 + 0.03 × GA	<0.001
Overweight or Obesity	3220	=0.95 − 0.01 × GA	0.775	=0.36 − 0.01 × GA	0.771
Maternal ethnicity					
Han	23,630	=0.52 + 0.05 × GA	<0.001	=0.20 + 0.02 × GA	<0.001
Minority	1662	=1.02 − 0.01 × GA	0.621	=0.39 − 0.01 × GA	0.681
Family income, CNY/year					
<200,000	18,004	=0.68 + 0.04 × GA	<0.001	=0.26 + 0.01 × GA	<0.001
≥200,000	7288	=0.46 + 0.05 × GA	<0.001	=0.17 + 0.02 × GA	<0.001
Maternal educational level					
College/university	17,502	=0.60 + 0.04 × GA	<0.001	=0.23 + 0.02 × GA	<0.001
High school or below	5148	=0.57 + 0.05 × GA	0.004	=0.22 + 0.02 × GA	0.004
Postgraduate	2642	=0.54 + 0.03 × GA	0.025	=0.20 + 0.01 × GA	0.045
Maternal physical activity					
Moderate	18,775	=0.47 + 0.05 × GA	<0.001	=0.18 + 0.02 × GA	<0.001
Light	6190	=1.06 + 0.01 × GA	0.636	=0.41 + 0.01 × GA	0.637
Active	327	=0.20 + 0.09 × GA	0.149	=0.06 + 0.04 × GA	0.128
Parity					
Multipara	12,576	=0.38 + 0.06 × GA	<0.001	=0.14 + 0.02 × GA	<0.001
Nullipara	12,716	=0.70 + 0.04 × GA	<0.001	=0.26 + 0.02 × GA	<0.001
Maternal smoking					
No	24,799	=0.65 + 0.04 × GA	<0.001	=0.20 + 0.02 × GA	<0.001
Yes	493	=0.48 + 0.05 × GA	<0.001	=0.30 + 0.04 × GA	0.058
Maternal secondhand smoke exposure					
No	22,251	=0.52 + 0.05 × GA	<0.001	=0.19 + 0.02 × GA	<0.001
Yes	3041	=0.78 + 0.04 × GA	0.064	=0.29 + 0.01 × GA	0.047
Maternal drinking					
No	24,590	=0.55 + 0.05 × GA	<0.001	=0.21 + 0.02 × GA	<0.001
Yes	702	=0.41 + 0.09 × GA	0.028	=0.15 + 0.03 × GA	0.028
Mode of conception					
Natural conception	24,412	=0.54 + 0.05 × GA	<0.001	=0.20 + 0.02 × GA	<0.001
Assisted reproduction	880	=0.83 + 0.01 × GA	0.785	=0.30 + 0.01 × GA	0.674
Morning sickness					
No	13,994	=0.44 + 0.07 × GA	<0.001	=0.16 + 0.03 × GA	<0.001
Yes	11,298	=0.53 + 0.04 × GA	0.002	=0.21 + 0.01 × GA	0.002
FA and/or multivitamin supplementation					
No	673	=1.13 − 0.04 × GA	0.322	=0.42 − 0.01 × GA	0.401
Yes	24,619	=0.53 + 0.05 × GA	<0.001	=0.21 + 0.02 × GA	<0.001

Abbreviations: BMI, body mass index; FA, folic acid; GA, gestational age.

**Table 4:** The association of maternal weight and BMI gain in early pregnancy with GA in normal or majority maternal status.

Maternal Age (Years)	*n*	Weight Gain (kg)	*p*	BMI Gain (kg/m^2^)	*p*
Total	7431	=0.28 + 0.08 × GA	<0.001	=0.10 + 0.03 × GA	<0.001
<35	6384	=0.30 + 0.08 × GA	<0.001	=0.11 + 0.03 × GA	<0.001
≥35	1047	=−0.02 + 0.14 × GA	<0.001	=−0.01 + 0.05 × GA	<0.001

Abbreviations: BMI, body mass index; GA, gestational age.

## Data Availability

The data presented in this study are available upon request from the corresponding author. All the data were obtained from the CBCS, and the authors are not permitted to share the data alone.

## List of Abbreviations

GA	Gestational age
BMI	Body mass index
CBCS	China birth cohort study
GWG	Gestational weight gain
CHD	Congenital heart disease
FA	Folic acid
MV	Multivitamins
WHO	World Health Organization

## Appendix A

**Table A1:** The percentiles of maternal weight gain at different gestational weeks.

Gestational Week (Weeks)	No	Mean	SD	Percentile (kg)										
				Max	99	95	99	75	50	25	10	5	1	Min
6~	1797	0.8	1.6	13.0	6.5	3.5	2.5	1.5	0.5	0.0	0.0	−1.0	−3.0	−6.0
7~	3226	0.9	1.7	15.0	6.0	4.0	3.0	2.0	0.9	0.0	−0.5	−1.0	−3.0	−12.0
8~	2992	1.0	2.1	15.0	10.0	4.0	3.0	2.0	1.0	0.0	−1.0	−2.0	−3.7	−10.0
9~	2183	1.0	2.2	15.0	9.0	4.8	3.0	2.0	1.0	0.0	−1.1	−2.0	−4.0	−7.1
10~	1705	0.9	2.5	15.0	10.0	5.0	3.4	2.0	1.0	0.0	−2.0	−3.0	−5.0	−9.0
11~	2716	1.0	2.6	15.0	9.8	5.0	4.0	2.0	1.0	0.0	−2.0	−3.0	−5.0	−10.5
12~	7082	1.1	2.7	15.0	9.0	5.0	4.0	2.0	1.0	0.0	−2.0	−3.0	−5.5	−10.0
13~	3591	1.3	2.9	15.0	11.0	6.0	5.0	3.0	1.0	0.0	−2.0	−3.3	−6.0	−12.0

**Table A2:** The percentiles of maternal BMI change at different gestational weeks.

Gestational Week (Weeks)	No	Mean	SD	Percentile (kg/m^2^)										
				Max	99	95	99	75	50	25	10	5	1	Min
6~	1797	0.32	0.65	5.34	2.49	1.31	0.94	0.53	0.20	0.00	0.00	−0.39	−1.08	−2.17
7~	3226	0.34	0.65	6.24	2.48	1.52	1.10	0.67	0.33	0.00	−0.20	−0.42	−1.12	−4.15
8~	2992	0.37	0.78	6.01	3.59	1.62	1.17	0.74	0.35	0.00	−0.39	−0.76	−1.41	−4.01
9~	2183	0.37	0.84	6.01	3.31	1.71	1.20	0.75	0.36	0.00	−0.44	−0.80	−1.56	−2.55
10~	1705	0.34	0.96	5.86	3.75	1.91	1.34	0.76	0.35	0.00	−0.75	−1.13	−1.98	−3.70
11~	2716	0.38	1.00	6.24	3.54	1.95	1.56	0.80	0.38	0.00	−0.78	−1.24	−2.00	−4.21
12~	7082	0.40	1.03	5.86	3.51	1.98	1.56	0.84	0.38	0.00	−0.80	−1.24	−2.14	−4.33
13~	3591	0.50	1.00	6.01	3.94	2.29	1.79	1.06	0.40	0.00	−0.78	−1.28	−2.20	−4.69
